# Patient experiences with oily skin: The qualitative development of content for two new patient reported outcome questionnaires

**DOI:** 10.1186/1477-7525-6-80

**Published:** 2008-10-16

**Authors:** Robert Arbuckle, Mark J Atkinson, Marci Clark, Linda Abetz, Jan Lohs, Ilka Kuhagen, Jane Harness, Zoe Draelos, Diane Thiboutot, Ulrike Blume-Peytavi, Kati Copley-Merriman

**Affiliations:** 1 Mapi Values Ltd, Adelphi Mill, Grimshaw Lane, Bollington, Cheshire, SK10 5JB, UK; 2 Family and Preventive Medicine, University of California, 5440 Morehouse Drive, Suite 3500, San Diego, CA 9212, USA; 3 RTI Health Solutions, 3005 Boardwalk Drive, Suite 105, Ann Arbor, MI 48108, USA; 4 Mapi Values Ltd, Adelphi Mill, Grimshaw Lane, Bollington, Cheshire, SK10 5JB, UK; 5 Lohs Research Group, 2170 West Freeman Road, Palatin, IL 60067, USA; 6 International Qualitative Marketing Research, Ludwig-Ganghoferstr. 33, Munchen D85551, Germany; 7 Pfizer Global R&D, 2800 Plymouth Road, Ann Arbor, MI 48105, USA; 8 Wake Forest University School of Medicine, 2444 North Main Street, High Point, NC 27262, USA; 9 Pennsylvania State University College of Medicine, 500 University Drive HU14, Hershey, PA 17033, USA; 10 charite – Universitatsmedizin Berlin, Chariteplatz 1, D 10117, Berlin, Germany; 11 RTI Health Solutions, 3005 Boardwalk Drive, Suite 105, Ann Arbor, MI 48108, USA

## Abstract

**Objective:**

To develop the content for two new patient reported outcome (PRO) measures to: a) assess the severity of symptoms; and b) the impact of facial skin oiliness on emotional wellbeing using qualitative data from face to face, and internet focus groups in Germany and the US.

**Methods:**

Using input from initial treatment satisfaction focus groups (n = 42), a review of relevant literature and expert clinicians (n = 3), a discussion guide was developed to guide qualitative inquiry using Internet focus groups (IFGs). IFGs were conducted with German (n = 26) and US (n = 28) sufferers of oily skin. Questionnaire items were generated using coded transcript data from the focus groups. Cognitive debriefing was conducted online with 42 participants and face to face with an additional five participants to assess the comprehension of the items.

**Results:**

There were equal numbers of male and female participants; mean age was 35.4 (SD 9.3) years. On average, participants had had oily skin for 15.2 years, and 74% (n = 40) reported having mild-moderate acne. Participants reported using visual, tactile and sensory (feel without touching their face) methods to evaluate the severity of facial oiliness. Oily facial skin had both an emotional and social impact, and was associated with feelings of unattractiveness, self-consciousness, embarrassment, irritation and frustration. Items were generated for a measure of oily skin severity (Oily Skin Self-Assessment Scale) and a measure of the impact of oily skin on emotional well-being (Oily Skin Impact Scale). Cognitive debriefing resulted in minor changes to the draft items and confirmed their face and content validity.

**Conclusion:**

The research provides insight into the experience of having oily skin and illustrates significant difficulties associated with the condition. Item content was developed for early versions of two PRO measures of the symptoms and emotional impact of oily facial skin. The psychometric validation of these measures reported elsewhere.

## Introduction

Oily skin, or seborrhea (ICD-9 code 706.3), [1-3] is a common condition affecting men and women, typically between puberty and about 60 years of age. It is characterized by the production of a quantity of sebum which is excessive for the age and sex of the individual.

Although excessive sebum production has minimal physical impact on body function, chronic oily skin can cause significant concern for people who have the condition.[4] Oily skin appears greasy and shiny, contributes to the development of acne, and is frequently accompanied by large pores on the face.[5] The consequences of excess sebum may be associated with adverse psychological and social effects resulting from associated acne and the appearance of skin oiliness and shine. Various studies have estimated 66% to 75% aged 15–20 years are affected.[5-7] Surveys have also found that sufferers feel ugly, uncomfortable or unkempt and annoyed by the condition.

Given that the experience of having oily skin is personal and subjective, validated patient-reported outcome (PRO) measures of severity of oily skin and its impact should be included in any clinical trials seeking to evaluate treatments for the condition. With this in mind, a review of the literature was conducted, but failed to identify any existing PRO measures specific to oily skin. However, since this work was completed, one oily skin specific PRO has been published, the Oily Skin Self Image Questionnaire (OSSIQ).[4] Differences and areas of convergence between the OSSIQ and the measures developed here are examined in the discussion of this article.

The literature review identified only one other dermatology instrument which included any oily skin symptom assessment questions (the Acne-Specific Health Related Quality of Life Instrument).[8-10] The health-related quality of life (HRQL) domains that were covered by acne and more general dermatology PROs did suggest a common set of concerns for patients with dermatologic conditions that might also be relevant to those with oily skin (e.g., Symptom Assessment & Impact, Emotional Distress, Coping & Functioning, Negative Image & Appearance, Self-Consciousness, Esteem & Confidence, Social Relationships & Stigma).

Due to the absence of validated PRO measures specific to the assessment of oily skin symptoms and its impact, an instrument development program was initiated. The aim was to develop PROs that would meet standards for development and validation recommended by regulatory authorities, and thus be acceptable as endpoints in clinical trials and as the basis of labeling or promotional claims. [11,12] This paper reports on the qualitative methods and findings from preliminary content discovery and validation of items used as the basis for two new PRO measures.

### Aims and conceptual basis for development of oily skin assessments

The following conceptual measurement objectives were developed based on the results of treatment satisfaction focus groups, a review of the research literature, and input from three dermatologists with expertise in oily skin and acne:

1. To identify the characteristics of skin oiliness that are commonly used by patients to know that their skin is oily,

2. To assess the different methods or techniques that individuals' use to self-assess these characteristics or symptoms,

3. To account for the biophysical and environmental conditions which impact or co-vary with self-assessed skin oiliness,

4. To evaluate the impact on patients' daily activities and emotional well-being.

These conceptual objectives were used to design a Discussion Guide for the Internet Focus Groups (IFGs).

## Methods

### Overview

**Figure 1:**
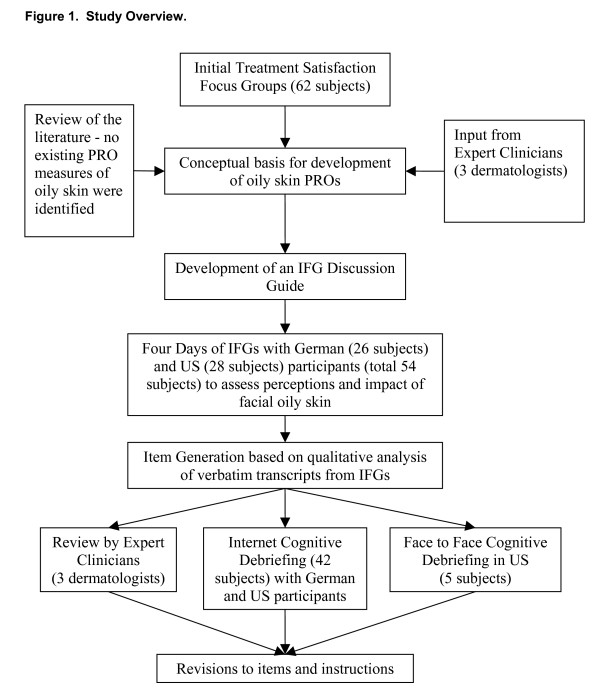
**Study Overview.**  This is a word file that provides a flow chart providing an overview of the study

An overview of the study methods is provided in Figure 1.

### Participants

• Initial treatment satisfaction focus groups were conducted with 62 adults with oily skin. In total, seven groups were conducted in the USA: six groups conducted in US English (n = 53); and one in US Spanish (n = 9).

• Exploratory IFGs were conducted involving a total of 54 participants with oily skin: 26 in Germany and 28 in the US.

• Of the 54 participants from the IFGs, 42 later participated in online cognitive debriefing, 8 in Germany and 24 in the USA. An additional 5 adults with oily skin in the US who had not participated in any of the focus groups participated in face to face cognitive debriefing interviews.

### Methods for participant recruitment

Participants were recruited through newspaper advertisements, the internet, and through GPs and dermatologists. A range of methods of recruitment was used to ensure the sample included both participants who were consulting with a physician about their oily skin, and others who were not.

The screening of candidates for participation specified that:

• all participants had to have self-perceived problems with oily skin

• all were actively trying to control their symptoms of oily skin, and

• all were interested in, or currently seeking, some form of treatment for their oily skin condition

In order to help assure the relevance of these content development activities to different gender, ethnic and acne subgroups, the IFG screening criteria was designed to ensure broad demographic representation (age, sex, ethnicity) and included some participants who had been treated by a physician for mild to moderate acne in the last 2 years.

### Initial face to face treatment satisfaction focus groups

Existing findings from face to face focus groups conducted with adults with oily skin to investigate treatment satisfaction with oily skin products were reviewed to identify potential domains that would be important to include in measures of facial oily skin and its impact.

### Internet focus group methodology

Four IFGs (two in Germany and two in the US) were conducted over four days during which participants answered sets of questions on a particular topic each day (based on the conceptual objectives listed above).

Within the password-protected IFGs, participants read and answered open-ended questions, responded to focused probes posted by the moderators, and responded to the opinions of other participants. Unlike traditional focus groups, respondents' worked on their own schedule at a convenient time each day, from home. Compared to a traditional focus group, the technology perhaps leads to responses being more completely considered, clearly presented, and candid. One native US English moderator and one German bilingual moderator facilitated the IFG sessions in the two countries in their native language. For more information about the application of IFG focus groups and this project in particular, readers are referred to a previous article by Atkinson et al (2006).[13]

### Cognitive debriefing methodology

Following development of the draft item pools, 42 (77.7%) of the 54 IFG participants (18 in Germany and 24 in the US) from the first round of focus groups participated in follow-up cognitive debriefing and importance rating activities. Following the initial round of IFGs it was observed that the IFG participants had relatively high educational levels. With that in mind, face to face cognitive debriefing was also conducted in five additional adults with oily skin in the US who had not participated in any of the online activities and were of relatively low educational status (only educated to high school level) to ensure the draft items would be understood by all adults. Conducting these interviews face to face also helped ensure findings were not being missed due to the IFG method.

In addition, expert clinicians were provided with the draft item pools and asked to review for any suggested modifications. During the cognitive debriefing participants were also asked to provide an importance rating for each item and answer a series of questions regarding their comprehension and interpretation of the questions (i.e., on-line cognitive debriefing). Results were used to refine the wording and instructions for items. Differences in importance ratings between the countries were noted for follow-up during later psychometric evaluation.

### Qualitative analysis

Following completion of the focus group sessions, the moderators carefully read each transcribed response made during the sessions and coded them into categories using a coding schedule. When a response contained information for which a coding category did not exist, a new one was created and flagged for follow-up during a 'harmonization discussion' with the other facilitator. Categories that appeared to be redundant or inadequately specified were also flagged.

Once cultural differences were identified and resolved, the coding schedule was finalized and a Content Frequency Analysis was performed based on the number of code endorsements (i.e., number of times mentioned by all participants) and the number of unique IFG participants coded to a particular topic category. Identification of the most commonly mentioned and coded topics formed the basis on which to design assessment items and create instructions to standardize the self-assessment procedure(s). Coding categories that were used by 15% or less of the overall participants were dropped. This quasi-qualitative approach to cross-cultural thematic analyses is similar to quantitative methods (i.e., cultural consensus analysis) based on the identification of cultural similarities and differences in values and perspectives.[14]

### Ethical Issues

The study was conducted following the principles outlined in the Declaration of Helsinki. Written informed consent was obtained from all study participants prior to their involvement in focus groups or patient interviews, in a manner that followed the US Health Insurance Portability and Accountability Act (HIPAA) guidelines. Due to the nature of oily skin, many people do not seek treatment from a physician. Therefore subjects were recruited through advertisements, and a physician confirmation of diagnosis was not required. Thus, as medical professionals were not involved in recruiting subjects, and given that the research was qualitative and observational (not involving any study interventions or medications), approval of the study from an institutional review board was not sought.

### Statistics

T-tests were used to compare mean importance ratings between the German and US samples. A significance level of p < 0.05 was used.

## Results

### Treatment Satisfaction Focus Group Results

The mean age of participants was 35.5 (SD 9.3) years (range: 19–61 years old), 73% were female, 47% were Caucasian, 25% Hispanic, 21% African American and 7% other; thus a range of ages and ethnicities were represented. Participants included those with self-rated mild (23%), moderate (52%) and severe (26%) oily skin.

The most common descriptions of oily skin were: "*shiny*" (n = 23), "*greasy*" (n = 17), "*oily*" (n = 7) and "*annoying*" (n = 6). Participants described the appearance of their oily skin as being "shiny" and oily "*like an oil refinery*". Shininess was a particular problem for participants with darker skin tones. Twenty four percent of women described having problems applying make-up. When asked to describe the feel of their oily skin participants used terms such as "*greasy*", "*clammy*", "*slimy*" and "*slippery*" and also referred to their skin feeling "*dirty*" or "*grimy*" and talked about a "*heavy*" feeling.

### Internet Focus Group Results

The demographic and clinical characteristics of the US and German internet focus groups are presented in Table 4. Mean age of participants was 35.4 years, the majority (94%) considered themselves to have moderate or severe oily skin, and 70% had at least some college education. Demographic and clinical characteristics of the two cultural samples were similar with two notable exceptions; a greater proportion of German participants reported they had been treated by a physician for their oily skin (69% vs 14%) and more females in Germany reported that their oily skin varied with hormonal variation (78% vs 38%).

**Table 4:** Internet focus group participant characteristics

**Demographic Characteristic**	**Total Sample (N = 54)**	**US Sample (N = 28)**	**German Sample (N = 26)**
Gender (f:m)	1:1	1:1.2	1:0.8

Mean Age	35.4 yrs (SD 9.3)	35.9 (SD 9.0)	34.8 (SD 9.7)

Mean years with condition	15.2 yrs (SD 9.4)	16.8 (SD 10.5)	13.4 (SD 8.0)

Moderate to Severe Oily Skin	94% (51/54)	89% (25/28)	100% (26/26)

Education (At least some college)	70% (38/54)	82% (23/28)	58% (15/26)

Married/CL	68% (37/54)	82% (23/28)	54% (14/26)

Self-Reported Acne	74% (40/54)	68% (19/28)	81% (21/26)

Oily skin varies with hormonal variation*	59% (16/27)	38% (5/13)	78% (11/14)

Oily scalp**	41% (11/27)	27% (4/15)	58% (7/12)

Treated by Physician for Oily Skin	41% (22/54)	14% (4/28)	69% (18/26)

Treated by Physician for Acne	41% (22/54)	18% (5/28)	65% (17/26)

Oily skin worsening with age	35% (19/54)	43% (12/28)	27% (7/26)

Polycystic Ovarian Syndrome*	29% (2/27)	8% (1/13)	7% (1/14)

Congenital Adrenal Hyperplasia	2% (1/54)	0% (0/28)	4% (1/26)

*females only

**males only

Participants were asked about the areas of the face (and scalp for balding men) where they had a problem with oily skin. The areas of the face most commonly chosen as being oily were the forehead (85%) and nose (83%) – oily skin was only a problem on the chin for 39% and the cheeks for 30% of the sample.

### Patient reported effects of oily skin

The effects of oily skin mentioned by the participants in the focus groups are summarised in Table 1.

#### Visual perception of oily skin

A shiny appearance was the most frequently reported effect of oily skin, reported by 96% (n = 52) of IFG participants:

*"I look in the mirror and the light is glistening off of my nose and forehead" *(US male participant – #19).

*"It reminds me of an oil slick that you would see on the pavement from a car dripping. It has a reflection to it. A glare." *(US female participant – # 10)

Seventy two percent 72% (n = 39) reported having pimples or blackheads.

#### Sensation of oily skin

When asked about the non-tactile sensation of oily skin, consistent with the treatment satisfaction focus groups, a large proportion of the participants (68%, n = 37) reported that their oily skin felt "*unclean*", "*dirty*" or "*grimy*". In addition 48% (n = 26) described a "*heavy*" feeling and 42% (n = 23) talked about discomfort generally. Other sensations described included itching, a feeling of oiliness or greasiness, the facial skin feeling hot or warm, and the feeling of having clogged pores or an additional layer of skin. Perhaps surprisingly only 35% (n = 19) described their skin as feeling "*oily*", "*slimy*" or "*greasy*"; although many participants described their skin feeling "*heavy*" or "*grimy*" in a manner that implied oiliness.

#### Tactile perception of oily skin

Participants typically reported that their skin felt oily or greasy to touch, and that their fingers also felt oily after they had touched their face:

"I rub my fingers on the sides of my nose & if my fingers are greasy it's oil. I sweat heavily & sweat drips off my face, the oil stays." (US male participant – # 22)

A few participants commented that on hot days the oil and sweat would mix and that was even more of a problem than their skin just being oily. Most participants were very clear that they could distinguish between oiliness and sweatiness.

"Sweat just runs down your body, like rain drops, but oil feels like butter – greasy" (US female participant – # 10)

**Table 1:** Patient-reported effects of skin oil

	**Total Sample (N = 54)**	**US Sample (N = 28)**	**German Sample (N = 26)**
**Visual Perception % (n)**			

Surface Shine (glow or skin reflection)	96% (52)	100% (28)	92% (24)

Moist/Wet Appearance	18% (10)	25% (7)	12% (3)

*Visual Consistency of Skin Surface*			

Pimples or Blackheads	72% (39)	78% (22)	65% (17)

Makeup runs	26% (14)	18% (5)	35% (9)

Pore Size	20% (11)	18% (5)	23% (6)

**Tactile Perception % (n)**			

Oily or slimy or greasy	35% (19)	54% (15)	15% (4)

Stickiness or Tackiness	31% (17)	7% (2)	58% (15)

Moisture (Sweat or Clamminess)	26% (14)	18% (5)	35% (9)

Dryness, Roughness, Bumpiness	26% (14)	25% (7)	27% (7)

*Slipperiness or Slickness*			

Due to Oiliness	61% (33)	32% (9)	92% (24)

Due to Wetness or Sweat	30% (16)	-	62% (16)

Depending on Temperature	30% (16)	-	62% (16)

**Sensory Perception (Sensory Feeling) % (n)**			

Unclean Sensation (dirtiness, griminess)	68% (37)	64% (18)	73% (19)

Heaviness (Heavy feeling)	48% (26)	61% (17)	35% (9)

Discomfort	42% (23)	25% (7)	62% (16)

Itching	35% (19)	21% (6)	50% (13)

Oily/Greasy Sensation	35% (19)	43% (12)	27% (7)

Skin Surface Temperature (Hot)	35% (19)	18% (5)	54% (14)

Skin Surface Temperature (Warm)	24% (13)	21% (6)	27% (7)

Clogged Pores	20% (11)	11% (3)	31% (8)

Feeling of Additional Layer	18% (10)	7% (2)	31% (8)

Sweating/Skin Moisture	17% (9)	14% (4)	19% (5)

#### Cultural differences

Harmonization discussions between the German and US moderators focused on a number of differences relating to the discussion of the Tactile and Sensory (non-tactile) feelings associated with skin oil. Cultural differences on these dimensions appeared to exist, with more German participants describing the skin as feeling (by touch) sticky or tacky (58% in Germany vs 7% in the USA) and more participants in the USA describing the oiliness or sliminess of their skin (54% in the USA vs 15% in Germany). Differences also seemed to exist on how participants attributed the slipperiness or slickness of their skin, with German more participants suggesting that perspiration and temperature have an impact. This observed difference, however, may have been due in part to differences between the moderators in terms of use of the Coding Schedule and the use of follow-up probes.

### Methods for assessment of oily skin

The methods participants used to assess their oily skin are summarised in Table 2. The majority of participants (83%, n = 45) assessed their facial skin oiliness and shininess by looking in the mirror.

*"Whenever I go to the bathroom and look in the mirror to touch up my make-up thru-out the day, my shiny skin is very noticeable" (US Female participant – #6)*.

When asked how they would assess the oiliness of their skin by touch, 52% (n = 28) talked about stroking their face and 35% (n = 19) examined the oil on their fingers after they had stroked their face.

"I can touch my face and tell that it is oily" (US male participant – #20)

"I touch my nose or chin and can see the oiliness in my fingers!" (US female participant – #2)

Just under half of the participants (42%, n = 23) reported blotting their face to assess its oiliness. Blotting was also frequently referred to as a method of managing their oily skin. Although only 24% (n = 13) of participants reported that they assessed the oiliness of their skin by non-tactile sensations, a greater number of participants did report that their skin felt "*heavy*" or "*dirty*". In some cases it seems the non-tactile feel of their skin would lead participants to touch their face for confirmation that it was oily. Several participants commented that the shininess, from when they looked in the mirror, the non-tactile feel and the feel to the touch all tended to happen together: if their skin had the sensation of being oily, they looked in the mirror and it would also appear shiny and feel oily to the touch.

"When I wake in the morning and my face feels heavy. I then touch my face and I can feel the grease. I look in the mirror and only confirm my suspicions, I have a shiny, oily face" (US Female participant – #7)

The endorsement frequency of certain Touch assessment categories among US IFG members was lower than for German IFG members. The moderators agreed that this was primarily due to differences in how the moderators probed the groups with respect to the frequency that they rubbed or stroked their skin when making a touch assessment. The same was also true for the use of moderator probes into the types of blotting materials used by participants. Supporting this explanation for observed differences, the observed endorsement frequencies did not differ between the two groups on either codes referring to the pressure of strokes, the rubbing of fingers together, or the blotting and visual inspection of the blotting paper.

**Table 2:** Methods for self-assessment of skin oil

	**Total Sample (N = 54)**	**US Sample (N = 28)**	**German Sample (N = 26)**
**Touch Assessment (Rub-Stroke) % (n)**			

Simple stroke	52% (28)	21% (6)	85% (22)

Number of strokes/rubs	39% (21)	18% (5)	62% (16)

Examining oil on fingers after touching	35% (19)	43% (12)	27% (7)

Pressure of stroke	31% (17)	32% (9)	31% (8)

Rubbing index finger and thumb	31% (17)	36% (10)	27% (7)

**Visual Assessment % (n)**			

Reflection in mirror	83% (45)	78% (22)	88% (23)

Location of mirror placement (Bathroom)	42% (23)	36% (10)	50% (13)

Blotting (and visual inspection of blot)	42% (23)	54% (15)	31% (8)

# of dabs before inspection	31% (17)	21% (6)	42% (11)

Frequency of self examination			

- 1 – 5 times per day	28% (15)	32% (9)	23% (6)

- 6 – 10 times per day	26% (14)	18% (5)	35% (9)

**Type of blotting material % (n)**			

- Tissue/Toilet paper	61% (33)	36% (10)	88% (23)

- Cleansing Tissues	30% (16)	7% (2)	54% (14)

- Paper towel	26% (14)	11% (3)	42% (11)

**Skin Sensations (non-touch) % (n)**	24% (13)	36% (10)	12% (3)

### Factors affecting self-assessment

Participants reported that a variety of factors influenced their perceptions of facial skin oiliness. The most common response given related to anxiety or stress levels (81%, n = 44), temperature (76%, n = 41), seasonal variation (68%, n = 37) and humidity (67%, n = 36). In addition, 59% (n = 16) female participants reported that their facial skin oiliness could be affected by hormonal variation.

### Emotional impact of oily skin

The  impact of oily skin on emotions and daily life is summarised in Table3. Oily skin participants reported feeling self-conscious and preoccupied about their oily skin – both were reported more frequently by German participants (73% (n = 19) and 85% (n = 22)) than by US participants (46% (13) and 28% (n = 8)). Participants also reported that they were worried about how others perceived their appearance, and that they frequently checked their oily skin and felt embarrassed around other people.

*"When I am around people or out in public then it becomes a bigger issue that makes me feel very self conscious about myself. Makes me feel dirty, like I am not a clean person (which I am!)" (US female participant – #6)*.

"It's embarrassing to go outand havemy face suddenly looking all greasy" (US male participant – #15)

"The feel becomes very annoying to me & that's veryimportant to me" (US male participant – #22)

Participants reported that their daily life was impacted by the need to constantly wash and blot their skin. Female participants talked of needing to apply face powder and frequently reapply their makeup. Eighteen percent (n = 10) of participants reported having to wash their face 6–15 times a day, 50% (n = 27) washed their face 3–5 times a day and 42% (n = 23) washed their face 1–2 times a day.

**Table 3:** Emotional impact of oily skin and impact on daily routine

	**Total Sample (N = 54)**	**US Sample (N = 28)**	**German Sample (N = 26)**
**Appearance and Social Impact % (n)**			

Perception of appearance	67% (36)	71% (20)	62% (16)

Self-consciousness	59% (32)	46% (13)	73% (19)

Social Confidence	18% (10)	18% (5)	19% (5)

**Distress/Interruption % (n)**			

Preoccupation appearance	56% (30)	28% (8)	85% (22)

Worry about need to manage condition	31% (17)	21% (6)	42% (11)

Frequency checking skin oiliness	18% (10)	14% (4)	23% (6)

**Impact on Daily life % (n)**			

Washing or Cleansing for oil control	65% (35)	75% (21)	54% (14)

Times of day when typically washing	44% (24)	46% (13)	42% (11)

Need to Blot	41% (22)	64% (18)	15% (4)

Apply Face Powder (females only)	52% (14)	38% (5)	64% (9)

Makeup (Re)Application (females only)	30% (8)	54% (7)	7% (1)

*Number of cleansings per day*			

- 1–2	42% (23)	36% (10)	50% (13)

- 3–5	50% (27)	39% (11)	62% (16)

- 6–15	18% (10)	11% (3)	27% (7)

*Effect on diet*			

- No Fast Food, No Rich Food	54% (29)	50% (14)	58% (15)

- No Chocolate, No Sweets	26% (14)	7% (2)	46% (12)

- Eat Healthy Foods, Eat More Fruit	26% (14)	36% (10)	15% (4)

### Item generation and design of the draft questionnaire

Based on the results of the qualitative content analysis summarized above, two broad assessment domains were identified, namely:

1. Self-assessment of skin oiliness and shine

a. Visual methods of assessment (looking in the mirror and looking at the oiliness of blotting paper)

b. Tactile methods of assessment (touching the face and feeling of fingers after stroking or rubbing the face)

c. Sensory feel methods (feeling of facial skin without touch)

2. Emotional impact of oily skin symptoms:

a. Annoyance and frustration related to having oily skin

b. Impact on body image and self esteem

c. Impact on social functioning

Prototype PRO items and instructions were drafted in these measurement domains based on the conceptual objectives described earlier.

**Figure 2:**
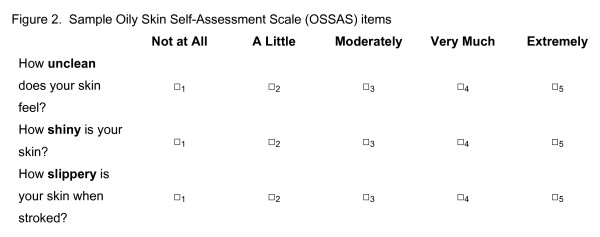
**Sample oil skin self-assessment scale items.**  This file is a word doc containing sample items from the OSSAS questionnaire

Item Pool 1 was focused on the Self assessment of skin oiliness and items in this pool were targeted for inclusion in the "Oily Skin Self-Assessment Scale (OSSAS)". They were designed for recording of 'spot' ratings made by feeling, touch and sight, either at a single point in time or over time. Example items are provided in Figure 2.

**Figure 3:**
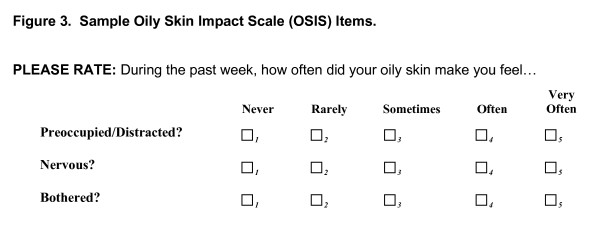
**Sample oily skin impact scale items.**  This file is a word document containing sample items from the OSIS questionnaire

Item Pool 2 was focused on measuring the emotional impact of oily skin and items in this pool were targeted for inclusion in the "Oily Skin Impact Scale" (OSIS). They were designed to assess the emotional impact and general level of distress associated with skin oiliness. Example items are provided in Figure 3.

New items used the natural wording and phraseology articulated by the US and Germany focus group participants, obtained from the transcripts. The items and instructions for the Oily Skin Self-Assessment Scale were tailored to the assessment methods that participants indicated they used. When making the non-tactile assessments participants were asked to answer the questions "without touching or looking at your skin"; when making the tactile assessments participants were asked to touch or stroke the most oily area of their face with a finger; when making the visual assessments participants were asked to look in the mirror.

Focus group results suggest that the reliability of self-assessment measures over time (reproducibility or generalizability) might be affected by a number of environmental and physiological conditions, not necessarily associated with sebum levels, but associated with individuals' perception of skin oiliness. Two approaches were used to help control for variation in these factors. The first was to standardize the conditions when individuals made their self-assessment through specific instructions. For example, subjects were requested to ensure they had not engaged in physical activity in the three hours prior to completing the questionnaire. The second approach was to include items which could be used as statistical covariates to control for factors that might result in otherwise unexplainable variation in self-perceived skin oiliness. These included items such as 'How hot does your skin feel?'

Given that oily skin is highly variable, for all of the OSSAS items, participants were asked to respond based on how their skin is "right now". In contrast, it was felt that for the OSIS, it would make more sense to participants to be asked to respond to questions about the emotional impact of oily skin based on a recall period of the past week. This decision was based upon input from both patients and expert clinicians in the field of dermatology.

### Results of importance ratings

The item importance ratings obtained during the on-line cognitive debriefing (conducted at the end of the IFGs) are provided in Table 4. **Internet focus group participant characteristics**

Table 5. Interestingly, the self-assessment summary items ("Overall,...") were given the highest importance ratings across IFG participants, likely due to their perceived relevance to all participants with any type of concern or experience.

**Table 5:** Importance ratings of draft OSSAS items by country

**OSSAS ITEMS (Rank Ordered by Mean Across All Groups)**	**All Groups Mean^++^**	**US Mean(SD)**	**German Mean (SD)**	**F value**	**P value**
**Overall, how oily does your skin look?**	**1.5**	**1.3 (0.4)**	**1.8 (0.7)**	**10.12**	0.00***

How shiny is your skin?	1.6	1.5 (0.9)	1.8 (0.8)	1.61	0.21

**Overall, how oily does the surface of your skin seem when touched?**	**1.6**	**1.4 (0.6)**	**1.8 (0.6)**	**4.48**	0.04*

**Overall, without touching or looking at your skin, how oily does your skin feel?**	**1.9**	**1.6 (0.8)**	**2.3 (0.9)**	**7.84**	0.01**

How oily was the blotting paper after pressing it once on your face?	1.9	1.8 (1.0)	2.0 (0.8)	1.10	0.30

How long has it been since you last washed your face?	2.0	1.8 (1.2)	2.1 (0.8)	1.00	0.32

How uncomfortable does your skin feel?	2.0	2.0 (1.2)	1.9 (0.8)	0.10	0.75

How unclean does your skin feel?	2.0	1.9 (1.0)	2.0 (1.0)	0.34	0.56

How sticky is your skin when touched?	2.0	2.3 (1.5)	1.8 (1.0)	1.68	0.20

How visible is your acne?	2.1	2.0 (1.2)	2.2 (1.5)	0.15	0.70

How visible are your pimples?	2.1	2.1 (1.2)	2.1 (1.1)	0.04	0.85

How moist does the skin surface look?	2.1	1.6 (1.0)	2.7 (1.1)	11.24	0.00***

How slippery is your skin when stroked?	2.1	1.7 (0.9)	2.5 (1.1)	8.46	0.01**

How oily do your fingers feel after stroking your skin?	2.1	1.7 (0.9)	2.6 (1.3)	7.66	0.01**

How heavy does your skin feel (i.e., like an additional layer or oily build-up was on the skin)?	2.2	1.9 (1.1)	2.5 (1.1)	3.33	0.08

How visible are your pimples or blackheads?	2.2	2.2 (1.3)	2.3 (1.3)	0.11	0.74

How moist is your skin when touched?	2.3	1.8 (1.1)	2.9 (1.3)	10.09	0.00***

How oily do your fingers or nails look after stroking your skin?	2.3	1.8 (1.0)	3.0 (1.3)	12.40	0.00***

How much do your pores feel, blocked or clogged?	2.5	2.2 (1.2)	2.9 (1.2)	4.31	0.04*

How enlarged do your skin pores look?	2.5	2.5 (1.4)	2.5 (1.1)	0.01	0.91

How flushed (red) is your skin?	2.7	2.6 (1.3)	2.8 (1.3)	0.30	0.59

How bumpy does the surface of your skin feel when stroked?	2.7	2.4 (1.3)	3.1 (1.3)	3.16	0.08

How itchy does your skin feel?	2.9	2.9 (1.5)	2.9 (1.3)	0.02	0.88

How hot does your skin feel?	2.9	2.9 (1.3)	2.9 (0.9)	0.04	0.85

How rough does the surface of your skin feel when stroked?	2.9	2.5 (1.4)	3.3 (1.3)	3.21	0.08

How uneven does your skin surface look due to flaking or dryness?	3.0	2.8 (1.4)	3.4 (1.0)	2.96	0.09

^++ ^1, Extremely important; 2, Very Important; 3, Important; 4, A Little Important; 5, Not important at all

* p < .05; ** p < .01; *** p < .001

Table 6 presents the mean importance ratings for the Oily Skin Impact Scale item pool for the total sample and by country, with differences between the two countries evaluated using a t-test. Importance ratings for German respondents were lower than for US respondents, particularly on the "Self-conscious", "embarrassed" and "discouraged" items, for which there were statistically significant differences between the two countries (p < 0.001, p < 0.02, p < 0.02, respectively). Interestingly, difficulties in translation of the concept of 'self-consciousness' were noted during linguistic validation of the OSIS item pool in German -the closest single term for 'self-consciousness' in German was back-translated 'insecure'. This, and the other two differences (Embarrassed and Discouraged), were noted for follow-up in later studies. The four items which were given the highest importance ratings ("Preoccupied/Distracted", "worried", "irritable" and "distressed" were rated highly in both the US and German IFGs, with no statistically significant differences between the two countries in the importance ratings for these items.

**Table 6:** Importance ratings for draft OSIS items by country

**Item**	**All Groups Mean^++^**	**US Mean (SD)**	**German Mean (SD)**	**F value**	**P Value**
Unattractive	1.6	1.7 (1.0)	1.4 (0.7)	1.37	0.25

Frustrated	2.0	1.9 (1.1)	2.1 (1.1)	0.50	0.49

Inconvenienced	2.1	2.1 (1.1)	2.1 (0.9)	0.01	0.94

Bothered	2.1	2.1 (1.0)	2.2 (1.0)	0.15	0.70

Embarrassed	2.1	1.8 (1.0)	2.5 (1.1)	5.43	0.02*

Nervous	2.2	2.5 (1.3)	2.0 (0.8)	2.24	0.14

Discouraged	2.2	1.9 (1.0)	2.7 (1.1)	5.83	0.02*

Annoyed	2.2	2.0 (1.1)	2.5 (0.9)	3.06	0.09

Disgusted	2.2	2.0 (1.1)	2.4 (1.3)	0.98	0.33

Self-conscious	2.2	1.6 (1.0)	2.8 (1.4)	10.75	0.00***

Preoccupied/Distracted	2.3	2.3 (1.0)	2.4 (0.9)	0.04	0.85

Worried	2.4	2.3 (1.2)	2.5 (1.2)	0.49	0.49

Irritable	2.4	2.4 (1.3)	2.4 (1.2)	0.03	0.86

Distressed	2.5	2.4 (1.1)	2.7 (1.1)	0.64	0.43

^++ ^1, Extremely important; 2, Very Important; 3, Important; 4, A Little Important; 5, Not important at all

* p < .05; ** p < .01; *** p < .001

### Results of cognitive debriefing and review and modifications to draft items and instructions

Feedback from the participants relating to the items and instructions was generally positive. Most items were well understood and considered by the participants to be relevant and important in relation to their oily skin. Participants generally understood the instructions and felt they would be able to answer the questions as part of a questionnaire assessing their oily skin. Results of the face to face cognitive debriefing were largely consistent with results from the internet focus group cognitive debriefing.

Changes were made to a few of the items based upon the comments of the cognitive debriefing participants and feedback from three expert clinicians who also reviewed the draft items. The following changes were made:

• An item relating to smell was removed as it was given a low importance rating and in the qualitative discussion was not generally felt to be important by participants of the focus groups. Smell had been mentioned by none of the focus group participants originally and had been added because the expert clinicians suggested it could be an issue

• An item asking about pimples and blackheads was split into two items as it was felt pimples and blackheads did not necessarily coexist

• An item which asked about the look of oil on fingers and nails was also split into two items as they were felt to be distinct concepts

• Items asking about feeling irritable, self-conscious and angry were added to the OSIS due to these terms being used by patients

## Discussion

The qualitative research reported here provides much needed insight into the experience of having oily skin from the patient perspective and the methods used by individuals to assess the oiliness of their skin.

The information given by participants relating to the impact of oily skin on their emotional wellbeing suggests that domains that are affected by having acne are also impacted by having oily skin. These include self-esteem, self-image, self consciousness and social functioning. In addition the maintenance of oily skin can be very time consuming requiring frequent cleansing and checking of the skin which can lead to feelings of frustration and annoyance.

The qualitative data from face to face focus groups in the US (the initial treatment satisfaction focus groups), internet focus groups in the US and Germany and input from opinion leader dermatologists were all taken into account in the development of items for two possible PRO questionnaires – a measure of facial oily skin severity (the Oily Skin Self-Assessment Scale) and a measure of the impact of oily skin on emotional wellbeing (the Oily Skin Impact Scale). Input was obtained from adults of both genders with a range of ages, ethnicities, cultural backgrounds (participants from both Germany and the US), geographical locations (thus ensuring the views of individuals in a range of climates were included) and self-perceived severities of oily skin. Input was also obtained from participants with and without comorbid acne. This is important as all of these factors might reasonably be expected to affect self-assessment of oily skin. In fact, few differences were noted across subgroups.

The item pool for the OSSAS that has resulted from this process consists of 26 items within two hypothesized domains of 'Perception' and 'Severity of symptoms' and within these 6 sub-domains. The item pool for the OSIS consists of 14 items within two domains of 'Self-concept' (3 items) and 'Emotional distress' (including the sub-domains of 'Anxiety' [7 items] and Annoyance [4 items]).

Since this work was completed, an instrument has been published that evaluates the emotional and behavioral impact of having oily skin – the Oily Skin Self Image Questionnaire (OSSIQ).[4] Both the OSIS and OSSIQ instruments focus on the emotional impact in terms of feeling self-conscious and unattractive; this consistency in content between the two instruments provides confirmation that the OSIS is focused on issues that are of importance to oily skin sufferers. However, the OSSIQ also includes a "behavior" domain which is focused on the impact of oily skin on a person's social life, and it does not include any items which ask about annoyance, irritability or frustration individuals experience related to having oily skin, as the OSIS does. As such, then, it could be argued that the two instruments provide complementary rather than redundant information. Which instrument is chosen for inclusion in a study will most likely be dependent on whether it is of greater value to assess 'annoyance' or impact on social functioning.

Following the development of a new PRO, it is important to assess its psychometric properties in the population of interest, to provide evidence of its ability to detect change over time and to attempt to define meaningful change in the PRO scores (often referred to as the minimal important difference). A psychometric validation study has been conducted in which the item pools and concurrent measures were administered to 202 subjects with oily skin; a manuscript reporting the psychometric validation study is in preparation. A limitation of the present study is that the focus group participants were all adults aged 18 and over. Therefore further focus groups and cognitive debriefing interviews have also been conducted with adolescents to ensure that the measures are applicable to adolescents as well (reported elsewhere, manuscript in preparation).

The current work demonstrates how the IFG methodology helped to overcome many of the geographical, linguistic and cultural challenges associated with face-to-face focus groups. The convenience of on-line IFGs facilitates participants in providing carefully considered responses that are well suited to qualitative analyses. Moreover, for a population such as this who are self-conscious about their appearance the relative anonymity of the IFGs provides an environment in which they will not feel judged by their appearance and so may be more at ease and able to provide more candid comments.[15]

Differences between cultural samples were found, both on content frequency results and on the importance ratings for draft items. These differences provide information about how to design instrumentation that is sensitive to cultural differences and which may perform better across different cultural setting. Generally, worded summary items were rated as more important in both countries than the more specifically worded items (see Atkinson MJ et al 2004[16] for a discussion of item content based on a continuum between Generality and Specificity of item content). As might be expected, more specifically worded items differed between cultures in ways that could not easily be explained by cultural differences in responses on the rating scales (see Atkinson MJ et al 2004[16] for a discussion of scaling differences between the US and Germany). Significant differences were observed in the rank ordering of specifically worded items on their importance to those with the condition. These differences have been noted for use during the next stages of PRO validation. While the cultural differences did not result in the exclusion of content from the broad item pool, the results did suggest that specific content areas should be checked for cultural specificity at each stage of instrument design and validation.

## Conclusion

Two possible PRO questionnaires – a measure of facial oily skin severity (the Oily Skin Self-Assessment Scale) and a measure of the impact of oily skin on emotional wellbeing (the Oily Skin Impact Scale) – were developed using a process conforming to current regulatory guidelines for the development of PRO questionnaires. Item development was based on input from a individuals with oily skin through face to face and internet focus groups. Moreover, participants included both genders, individuals with and without acne and a range of oily skin severities, ethnicities and ages. Such a comprehensive instrument development process resulted in two pools of items that should be relevant to oily skin sufferers. The next step is psychometric validation of these item pools.

## Competing interests

Mark Atkinson, Marci Clark, Kati Copley-Merriman and Jane Harness were employees of Pfizer when the project was performed. All other authors were contracted by Pfizer as consultants to work on this project.

## Authors' contributions

MA conceived of and designed the study. LA, DT, UB-P, ZD, RA, and KC all also contributed to the design and planning of the study. RA and LA wrote the treatment satisfaction focus group guide, and analyzed the treatment satisfaction focus groups. MA, RA, and LA wrote the internet focus group discussion guide, including both exploratory and cognitive debriefing phases. DT, UB-P, ZD and KC all reviewed and provided input on the internet discussion guide. JL and IK moderated the internet focus groups. JL, IK and MA performed the analysis of the internet focus groups and cognitive debriefing. MA, LA and RA drafted the questionnaire items. DT, UB-P, ZD, MC, JH and KC all reviewed and provided interpretation and comments on the internet focus group results and the draft items.

RA, LA and MC wrote the face to face cognitive debriefing interview guide, RA performed the cognitive debriefing analysis and all authors reviewed and commented on the results of the face to face cognitive debriefing. RA and MA wrote up the results and wrote the first draft of the manuscript. All authors reviewed the manuscript and provided input on later drafts.
